# The effects of social capital on entrepreneurial resilience of SME from China: A moderated mediation model of entrepreneurial passion and Confucian traditional golden-mean thinking

**DOI:** 10.3389/fpsyg.2022.961824

**Published:** 2022-10-03

**Authors:** Tingting Shan, Xiaoya Tian

**Affiliations:** School of Management, Nanjing University of Posts and Telecommunications, Nanjing, China

**Keywords:** social capital, entrepreneurial passion, Confucian traditional golden-mean thinking, entrepreneurial resilience, moderated mediation model

## Abstract

Small and medium-sized startups play a crucial role in a country's sustainable development. SMEs are at an early development stage, which means weaker institutional norms and successful paradigms, tending to suffer from exceptionally high failure rates because of many constraints, including a lack of resources and credit to access the core information. The higher the environmental uncertainty, the more likely that new start-ups rely on all kinds of social links for acquiring resources. As a critical ability to withstand and overcome obstacles, entrepreneurial resilience is an essential personal characteristic to ensure the sustainability of new ventures. The purpose of this study is to investigate the internal mechanism through which SME entrepreneurs' social capital affects entrepreneurial resilience. To reveal the internal mechanism, we propose a moderated and mediation model. Using survey data from SEM entrepreneurs in China, hierarchical regression analysis and bootstrapping model are adapted to test and verify mediation and moderation effects. The results show that social capital indeed positively influences entrepreneurial resilience directly and partly through the mediating effect of harmonious entrepreneurial passion and obsessive entrepreneurial passion. Moreover, findings suggest golden-mean thinking negatively moderates the relationship between obsessive entrepreneurial passion and entrepreneurial resilience yet positively moderates the relationship between social capital and entrepreneurial resilience. Based on these findings, we conclude that entrepreneurial resilience may be achieved more effectively through the complex internal process of social capital, entrepreneurial passion, and golden-mean thinking. Finally, the study proposes the theoretical and practical implications and suggestions for follow-up research.

## Introduction

Research related to new start-ups has recently arisen, notably after the venture boom of the 2000s (Song et al., 2017). China is currently experiencing a grip of venture fever. Expansion of the new start-ups in China's economy is a crucial and decisive driver of sustainable economic growth (Duchek, 2016). Among these new ventures, most are small and medium-sized start-ups (Colombo et al., 2010). According to China's statistics bureau, small and medium- sized enterprises accounted for 87.2% of all new start-ups in 2020. Here small and medium-sized enterprises in China are defined as “a separate distinct entity including cooperative enterprises and non-governmental organizations managed by one or more owners (Pietra, 2012).” The term “SME” is generally used to mean small and medium-sized enterprises. The indicators used to identify SMEs in Chin include the number of employees and the gross asset value of the whole entity (Bullough and Renko, 2013). The study defines SMEs as new start-ups whose number of employees is <250 (Parida et al., 2012). An SME entrepreneur is defined as the founder of a new start-up with <250 employees in China (Zhang et al., 2017). In China, SMEs are becoming increasingly important in the highly uncertain and competitive environment (Kai et al., 2021). Although there is such a large number of SMEs in China, not every SME can achieve success. Compared to large-scale and intra-company entrepreneurship, SMEs confront a relatively unsupportive environment which means more resource constraints and higher failure rates (Sripongpun, 2021; Zhao et al., 2021).

Issues related to failures of SMEs mainly focused on external causes. Due to the inherent weakness, most research attributes the failures of SMEs to external factors, including bad financial performance (Azavedo and Gogatz, 2021) and the lack of seed capital raised (Brouthers and Nakos, 2010). However, the intrinsic causes of the failure of SMEs are ignored. Inherent reason is closed related to the personality of an entrepreneur. Entrepreneurs are the core of the management and development of new start-ups. In particular, the psychological and behaviorally continuity of the individuals who start a business can help enterprises in the volatile environment of uncertainty to overcome difficulties, thus maintaining sustainable growth of the new start-ups (Uy et al., 2017). Therefore, it is meaningful and beneficial to investigate how to cultivate and promote entrepreneurial resilience.

Some studies have confirmed the importance of social links and social support to ensure the sustainability of individuals' mental levels (Ismail et al., 2015; Stroe et al., 2017). According to the interviews, we found that many entrepreneurs consider the social networks to be the key to the sustainable development of SMEs. Based on network theory, the social network plays a vital role in developing new start-ups (Cardon et al., 2017). However, the internal mechanism isn't precise now. Meaning-construction theory indicates that social capital or social links embedded in different cultural contexts is a core and valuable source which brings identity meaning to the mind of individuals (Onyx and Bullen, 2016). This kind of “identity meaning” gives them a positive emotional experience (Wang et al., 2022). In entrepreneurial activity, the essence of this positive emotion is entrepreneurial passion (Azavedo and Gogatz, 2021). But little research is available on the mediating effects of entrepreneurial passion. Therefore, this study explicitly investigates how passion mediates between social capital and resilience.

While significant progress has been made in studying the effects of social capital on entrepreneurs in developing countries, there are four essential gaps: Firstly, insufficient depth of content. In the face of the increasing scale of SME entrepreneurs, how to effectively transform their social network capital into entrepreneurial resilience is a crucial issue to enhance the effectiveness of SME entrepreneurship further. Previous studies have mainly explained the effect of social capital from the perspectives of knowledge spillover and social learning. Still, they have not given a relatively complete explanation and path description of the transformation mechanism. The extensive entrepreneurial network capital can help the entrepreneurs of small and medium-sized enterprises open up a new emotional value range, and positively affecting their value creation, competitive advantage, and organizational adaptability, thus promoting entrepreneurial resilience. In addition, most of the research results lack dialogue with the classical theory, which leads to the deficiency of the depth of analysis and the integrity of the theoretical framework. Secondly, the analytical hierarchy is not clear. The theory of meaning construction states that social capital or social connections embedded in different cultural contexts are the core and valuable source of identity meaning for individuals (Cattell, 2021). This “Sense of identity” gives them positive emotional experiences (Gargiulo and Benassi, 2010). The essence of this positive mood in entrepreneurial activity is entrepreneurial zeal (Wang et al., 2022). However, there are few studies on the intermediary effect of entrepreneurial zeal. The existing studies do not distinguish the levels of entrepreneurial passion, which leads to the fragmentation of the research levels, and the conclusion of research is difficult to be integrated into a systematic understanding. Thirdly, the theoretical perspective does not match the attributes of the research object. The existing research does not include the characteristics of SME entrepreneurs in the analysis and modeling, in essence, it is still regarded as a general entrepreneur. Based on the concept of entrepreneur of small and medium-sized enterprises in developing countries, it is necessary to conduct in-depth research on the characteristics of “Insufficient resources support,” “Lack of emotional support,” and “High failure rate,” these two characteristics correspond to the core variables of this paper (entrepreneurial network capital, entrepreneurial passion, entrepreneurial resilience). Fourthly, the context is not closely related. Some scholars point out that the effect of social capital is regulated by organizational context and individual characteristics. However, the existing literature does not include it in the study of the functional boundary of social capital, leading to inconsistent conclusions (positive, negative, not significant) on the direction of the impact of social capital on entrepreneurial resilience.

Although the concept of the golden mean can be found in the classical theory behind the golden mean, there is a unique Chinese philosophy of life and world outlook, involving self, interpersonal, and all aspects of doing things, China with its cultural background is more able to conduct an in-depth discussion of the golden mean. In the Chinese context, the doctrine of the mean is the core element of Chinese cultural psychology to cope with the challenges of diversity and diversification. Because the mode of thinking can exist independently from real life and has the possibility of being passed down from generation to generation, this article chooses the word “Golden mean thinking” to put this ancient cultural concept into modern research.

The purpose of this study is to verify the mediation effect of entrepreneurial passion between the entrepreneur's social capital and entrepreneurial resilience and the moderation effect of Confucian traditional golden-mean thinking. The next sector of this article will present a review of relevant studies and hypotheses extracted from them. Then the methodology, including the sample, measurement of critical variables, and statistical models, will be discussed. Finally, the analysis/empirical results will be revealed, and discussed and the theoretical and practical implications will be proposed. This article will be ended with a conclusion.

## Theoretical basis and hypothesis

### Social capital

Bourdieu (1986) believed that social capital is an enterprise whose members join together to create opportunities with more significant potential than competitors or have an advantage over competitors in the market. Entrepreneur social capital helps entrepreneurs identify opportunities, use resources effectively, and make the best decisions (Campos, 2016; Lamari, 2022). Whether an enterprise has an advantage over its competitors depends largely on social capital. Entrepreneurs in structural holes have a more tremendous advantage than other positions because they can get more practical information and scarce resources first (Cardon et al., 2016). Social capital is a kind of scarce social resource existing in the network of entrepreneur relations. The existence of social capital is not static but has a cumulative effect (Moran, 2015). Baron regard entrepreneur social capital as a collection of relational resources that can help entrepreneurs solve all kinds of problems faced by the start-up stage of new ventures (Cardon et al., 2017). Entrepreneurs should actively integrate and develop their social capital to promote the improvement of entrepreneurial performance (Breugst et al., 2012). The richer the social capital of entrepreneurs is, the stronger the development strategy can be put forward for the development of enterprises, thus promoting entrepreneurial activities and having a positive impact on entrepreneurial performance (Caliendo and Kritikos, 2019). Social capital can be divided into homogeneous social capital and heterogeneous social capital. Both types of social capital can improve the improvement of entrepreneurial performance (Cardon et al., 2013). Entrepreneurs' personal capital is especially important for the survival and development of enterprises (Rahayu et al., 2022). Entrepreneurs should actively cultivate social capital, obtain scarce resources through social network, and further improve the entrepreneurial performance of enterprises (Ngoma et al., 2021). However, other studies have found that there are other relations between entrepreneurs' social capital and entrepreneurial performance. Too much social capital of entrepreneurs will cause enterprises to need more input to maintain social relations, which may restrict the generation of innovative ideas and innovative decisions of enterprises, thus restricting the growth of enterprises (Richardson et al., 2022). Therefore, at present, the mechanism of the effect of venture capital on entrepreneurial output is not clear. Therefore, it is necessary to conduct an in-depth study on the role of social capital in entrepreneurial passion, entrepreneurial resilience, and entrepreneurial performance.

### Entrepreneurial resilience

The formation process of entrepreneurial resilience is “ironing out” the risks and uncertainties brought by internal and external shocks (Haddoud et al., 2022). The focus of entrepreneurial resilience research has been shifted with the change in crises (Pascucci et al., 2021). Flexibility emphasizes “rapid adaptation to environmental changes” (Schepers et al., 2021). Agility focuses on “quickly identifying opportunities (Mignenan, 2021), changing direction (Iyengar et al., 2021), and avoiding conflict (Branicki et al., 2018).” Robustness is more concerned with maintaining function in the face of interference (Li et al., 2022). Entrepreneurial resilience emphasizes the ability of an SME entrepreneur to recover and rebound under the impact of adverse events and grow against the trend in the process of reflection and improvement (Lafuente et al., 2019). Although flexibility, agility, and robustness are structurally similar to resilience, the former three are necessary to deal with everyday problems and changes, the resilience is seen as an essential success factor in coping with unexpected threats and responding to crisis changes (Nsereko, 2020).

Entrepreneurial resilience is an extension of the concept of resilience in ecology and physics (Zhang et al., 2017), used initially to measure a system's ability to recover after a disaster. The multidimensional and multi-level characteristics of entrepreneurial resilience make scholars' interpretation of its concept gradually increase. Representative literature, such as Holling (1973), pointed out from the perspective of ecology that entrepreneurial resilience is to re-establish a balanced adaptive behavior between the organization and the existing environment. Kantur (2015) constructed the conceptual framework of entrepreneurial resilience from the perspective of systems science and believed that entrepreneurial resilience results from the combined effects of the four elements of corporate position perception, situational integration, strategy formulation, and execution. From the perspective of crisis management, The Domestic scholar Shang et al. (2010) defined entrepreneurial resilience as the ability of an organization to reconstruct organizational resources, processes, and relationships in a situation, recover quickly from the crisis and realize countertrend growth by using the problem. It is not hard to find that despite the diverse perspectives of scholars worldwide, the view that the entrepreneurial resilience of a SME entrepreneur is essentially a “capability” has been widely accepted. In this study, the entrepreneurial resilience of the founder of a new start-up is considered a kind of intrinsic capability. From the perspective of capacity, entrepreneurial resilience refers to the incremental ability of a SME entrepreneur to predict the next crisis and adjust its strategy rapidly.

### Mediating effect of entrepreneurial passion

In this sector, we propose a mediation mechanism of entrepreneurial passion between social capital and entrepreneurial resilience. There are four steps to building this mediating effect model (Burt, 1997). Firstly, the positive link between predictor (social capital) and work (entrepreneurial resilience) should be verified (step1). Secondly, the positive relationship between mediator (entrepreneurial passion) and outcome (entrepreneurial resilience) should be satisfied, and the associations between predictor and mediator should also be tested positive (step2 and step3). Finally, the mediation effect is established if the strength of the step1 is significantly reduced while controlling the mediator (step4).

In a rapidly changing environment, new ventures are usually born with “new entry defects” and “small size defects,” and thus often confronted with severe resource constraints (Pelling and High, 2015) and information asymmetry, which function as inhibitions of venture performance. For the funders of the SMEs (small and medium-sized enterprises), building and leveraging social networks is more critical for the sustainable growth and success of the new start-ups (Dai et al., 2015; Baker, 2020). Social networking is a crucial channel for them to access essential information and require valuable resources (Wasko and Faraj, 2015).

In implementing entrepreneurship, information asymmetry and resource constrains could be noticeably mitigated when the individual embeds the social network deeply (Batjargal, 2016). The higher the network reliance, the more trust he will get from the social network. When the entrepreneurs and the information providers rely on each other deeply, the numbers in the networks are more likely to share vital and valuable information with those they trust. The information from external sources enables an entrepreneur to be less prone to adversities by facilitating the development of individual cognition (Thompson and Zang, 2018). As mentioned earlier, new ventures especially SMEs in China's transitional economy, frequently face quite a lot of resource constraints. An entrepreneur must consistently need to seek heterogeneous external resources beyond exciting resources to overcome homogenizing risk (Lehtonen, 2014). The founders of small and medium-sized enterprises need to acquire heterogeneous mental resources through stable and extensive social capital networks to enhance the organizational cognitive level and entrepreneurial orientation of the enterprises, breakthrough path-dependent risks, thus strengthen the innovation ability, maintain the sustained and steady development of enterprises (Woolcock, 1998). For example, networking between technology-based ventures and their key customers helps startups accumulate essential knowledge, which promotes the development of new products (Thompson and Zang, 2018).

Previous studies argue that an entrepreneur's network determines financial capital acquisition. Since one of the most significant risks for start-ups is cash flow-chain-broken, access to venture capital is critical (Robert and Steel, 2012; Ellison et al., 2020). Social capital networks help entrepreneurs to get financial capital to avoid falling into a cash-flow trap. Entrepreneurs who frequently behave in a trustworthy manner in social networks would receive additional investment from angel investors (Micu et al., 2018). The psychological fear of entrepreneurial failure is also the critical obstacle that leads entrepreneurs to give up entrepreneurial behavior. When these entrepreneurs have deep social capital, they will get a strong perception of entrepreneurial support and reduce the fear of failure risk, to keep their positive morale, the whole heart, and soul into entrepreneurial activities, thus to ensure the continuity of entrepreneurial activities (Dai et al., 2015; Stephen and Philip, 2017; Baker, 2020).

In addition to their weak entrepreneurial ability, lack of start-up capital, low entrepreneurial mental energy, and difficulty in obtaining entrepreneurial information, new start-ups also have a fatal weakness (Ratelle et al., 2004; Garg et al., 2011; Kadile and Biraglia, 2017). That is, they cannot establish a high-quality transaction network with low transaction costs in the short term (Yli-Renko et al., 2012). It is noticeable that a new startup tries to overcome the liabilities associated with its newness through networking (Granovetter, 2013; Narayan and Cassidy, 2021). Social capital allows them quickly into the trading network that they do not possess so that they can get the trading channels, which will become the core competitive advantage of enterprises. It is of great benefit to the sustainable development of enterprises (Markman and Baron, 2014; Thompson et al., 2020). Therefore, this paper proposes the following hypotheses:

**Hypothesis 1:** Social capital has a positive impact on entrepreneurial resilience.

To a great extent, the organizational will of the new start-ups are influenced by the individual characteristics of the entrepreneur (Batjargal, 2016). Entrepreneurial passion, as the most important trait on the emotional level, are the inherent individual characteristic. As a critical affective emotional factor, entrepreneurial passion was first proposed by Coleman (2020). Subsequently, entrepreneurial passion, a concept in psychology, was recognized by more and more scholars in the field of entrepreneurship. At the initial stage of starting a business, the founders of SMEs are more likely to encounter financing difficulties and resource shortages. It is easy for them to lose confidence and persistence in implementing new start-ups (Xie and Chen, 2014; Org, 2017). Previous research has shown that the entrepreneurial process is an emotional journey. Passion is a positive emotion that entrepreneurs hold toward various challenges. The social cognitive theory holds that social networking and social environmental factors can affect entrepreneurial emotional aspects through cognitive (Azazz and Elshaer, 2022). Relevant research shows that entrepreneurial support from the external environment has a non-negligible role in stimulating entrepreneurial passion (Baron and Markman, 2013; Parr, 2019).

Some scholars examine the influence and mechanism of entrepreneurial passion by empirical methods (Huang, 2017). It is found that entrepreneurs with high passion are more tending to achieve excellent performance, including promoting entrepreneurial effectiveness, and improving corporate business outcomes (Liao and Welsch, 2020). Some studies show that entrepreneurial passion plays a mediating role in the effect of policy support on entrepreneurial failure learning. Other studies have studied the negative environmental impact of entrepreneurs' exit intensions from a reverse perspective, confirming the mediating role of entrepreneurial passion (Cardon et al., 2019; Coppens and Knockaert, 2022). However, at this time, the literature only regarded entrepreneurial passion as a whole idea, ignoring the various dimensions within the concept of entrepreneurial passion.

With the deepening of the research, scholars gradually realize that the exploration and refinement of different dimensions within entrepreneurial passion will help reconcile the differences in research conclusions and make the research more valuable and targeted. There are different categories of entrepreneurial passion. Some scholars divide entrepreneurial passion into individual passion, team passion, and organizational passion (Jin and Jia, 2017; Wright and Mosey, 2020). Some scholars divided the entrepreneurial passion into control passion and coordination passion. Other scholars divide entrepreneurial passion into two categories: harmonious entrepreneurial passion and obsessive entrepreneurial passion (Maureen and Russell, 2013; Wasko and Faraj, 2015; Chang and Chuang, 2021; Clayton and Feldman, 2021).

The influence mechanism of social capital on the two kinds of passion is different. The influence of social capital on harmonious passion is realized by arousing the entrepreneur's genuine interest and love for the business he is engaged in. When an entrepreneur is embedded in a high-quality and broad-based social network, it partly means that he has deep social capital (Batjargal and Liu, 2012; Li, 2020; Azazz and Elshaer, 2022). Deep ties to government agencies, research institutions, and upstream and downstream partnerships enable the entrepreneur to learn, imitate, and worship others. The entrepreneur draws a lot of precious cognitive resources from the social capital, thus to improving his cognition and finding his interest more easily (Westlund and Bolton, 2013; Singh et al., 2021; Sobczak and Głuszczuk, 2022). Social capital helps entrepreneurs to combine their talents and aspirations which are rooted in themselves, inspire a harmonious entrepreneurial passion (Yu-Feng and Neng-Quan, 2015; Feldman, 2021; Qian et al., 2021; Backhaus et al., 2022).

Different from the generating process of harmonious entrepreneurial passion, the process of social capital arousing obsessive entrepreneurial passion is to endow the meaning of an entrepreneur's identity (Cabras and Mount, 2015; Zahra et al., 2019; Tian et al., 2022). According to the theory of meaning construction, human beings are social animals, and social networks endow other individuals with different identities in different contexts which have different meanings. Emotion originates from meaning. Social capital can strengthen the process of meaning construction (Bullough and Renko, 2013; Luo et al., 2022). The Passion comes from the explicit and implicit contract relationship between the entrepreneur and the external environment, which is also embodied in the “Root.” The more external contracts the entrepreneur has, the more diversified the roles he assumes. From the perspective of Social Relevance Theory, to meet the requirements of these contracts, entrepreneurs of small and medium-sized enterprises are more inclined to actively seek contract implementation opportunities, and to prove themselves, to construct meaning, which will strengthen the role of entrepreneurial passion. The more social capital an entrepreneur has, and the more social networks he embeds, the more various identities he has. The multiple identities will give him different identities (Shane and Cable, 2012). Obsessive entrepreneurial passion is stimulated in the process of realizing the meaning of identity. For example, a man has multiple roles as father, son, wife, and company executive. Social networks give him different social identities, and social identities give him identity meanings. He tries to fulfill his obligations as a father, son, wife, and corporate executive, even if it's not from his genuine love and affection (Abubakars and Garba, 2021; Metz et al., 2022; Moraisdasilva et al., 2022).

Therefore, this paper proposes the following hypotheses:

**Hypothesis 2**: Social capital positively influences Entrepreneurial Passion.
H2a: Social capital positively influences harmonious entrepreneurial passion.
H2b: Social capital positively influences obsessive entrepreneurial passion.

Most scholars agree on the positive effect of harmonious passion on the persistence of an entrepreneur's behavior and psychology. Entrepreneurs with a high entrepreneurial passion are good at turning environmental uncertainties into grand plans for seizing opportunities to succeed, thus improving corporate performance. However, it is not clear whether obsessive entrepreneurial passion has a positive effect on entrepreneurial resilience (Collewaert et al., 2016; Sulistiowati, 2019; Pei, 2021). The issue is still controversial. Some scholars hold if compulsive passion exceeds a certain threshold, it will make the entrepreneur's psychology collapse and cause physical exhaustion. Instead, it forces entrepreneurs to opt out of entrepreneurial activities (Bjornskov, 2012). Others insist that even if the compulsion-driven entrepreneurial behavior is not motivated by an individual intrinsic love or interest, it is bound up with social identity, the incentive effect on individual entrepreneur's behavior persistence is stronger and more significant than harmonious passion (Muscio and Vallanti, 2022). The arguments originate from a different research background, research objects, and research situations (Crick and Crick, 2016; Cardon et al., 2017). The object of this study is more focused on small and medium-sized enterprise (SME) entrepreneurs in China's transitional economy.

Entrepreneurial Passion plays a heterogeneous effect in the four steps of “Ignorant optimism,” “Informed pessimism,” “Subversion of values,” and “Informed optimism” (Adomako and Ahsan, 2022). From the second stage onwards, the small and medium-sized enterprise entrepreneurs fall into the most challenging state of persistence (Lucas et al., 2016; Milanova and Naydenova, 2022). Some encounter the failure of starting a business. Some have made achievements, but their steps are complicated, encountering bottlenecks. And it is tough to maintain the initial state of excitement (Li et al., 2017; Keoy et al., 2022). The obsessive entrepreneurial passion can provide adequate support for the future entrepreneurial behavior of SME entrepreneurs, thus helping to reduce the fear of SME entrepreneurs facing failure and enhance their entrepreneurial resilience in a crisis (Tahsildari et al., 2014; Micu et al., 2018). This study argues that both kinds of entrepreneurial passion (harmonious entrepreneurial passion and obsessive entrepreneurial passion) can benefit entrepreneurial resilience. Here propose the following hypotheses:

**Hypothesis 3:** Entrepreneurial Passion positively influences entrepreneurial resilience.
H3a: Harmonious entrepreneurial passion positively influences entrepreneurial resilience.
H3b: Obsessive entrepreneurial passion positively influences entrepreneurial resilience.

Entrepreneurial Passion is the specific emotion in the entrepreneurial activity, which refers to the intense and positive emotional experience of the entrepreneur through the entrepreneurial practice and the recognition of the meaningful and unique identity of the entrepreneur (Bird, 1998). This definition clarifies the two connotations of entrepreneurial passion: self-identity and strong positive emotions (Honig and Davidsson, 2020). In other words, entrepreneurial passion is not a general emotion, it needs to be stimulated by activities that have the meaning of entrepreneur identity. Individuals will experience entrepreneurial passion when they perceive their own entrepreneur identity from some activities (Huggins and Thompson, 2015; Jaskiewicz et al., 2015). In the process of entrepreneurship, on the one hand, entrepreneurs' social capital provides more diversified cognitive resources and more heterogeneous and high-quality information resources for small and medium-sized start-ups, which helps entrepreneurs overcome resource constraints and identify entrepreneurial opportunities more effectively, to enhance the ability of innovation, ease the fear of entrepreneurial failure, which gives entrepreneurs more confidence to overcome difficulties, mobilize the positive emotions of entrepreneur (Hao et al., 2015; Miles, 2016; Reza and Daria, 2022); on the other hand, the positive value information conveyed by social capital makes entrepreneurs with multiple roles evaluate the identity of “Entrepreneur” and enhance the identity of entrepreneurs (Khurana et al., 2021; Hu et al., 2022). This will significantly enhance the psychological and behavioral persistence of entrepreneurs, and entrepreneurial resilience in this process is enhanced (Hao et al., 2020; Santoro et al., 2020). Therefore, this paper proposes the following hypotheses:

**Hypothesis 4:** Entrepreneurial Passion plays a mediating role between social capital and entrepreneurial resilience.
H4a: Harmonious entrepreneurial passion plays a mediating role between social capital and entrepreneurial resilience.
H4b: Obsessive entrepreneurial passion plays a mediating role between social capital and entrepreneurial resilience.

### Moderating effect of Confucian traditional golden-mean thinking

A moderation effect occurs when there is a third variable between the independent and dependent variables. This third variable is called a moderator, which changes the strength or direction of the link between the two variables. Moderators are generally introduced in previous studies when the relations are inconsistent. According to earlier investigations, the relation between entrepreneurial passion (harmonious entrepreneurial passion and obsessive entrepreneurial passion) and entrepreneurial resilience appears elusive. Some studies insist that high and intense entrepreneurial passion enhances the sustained investment in the psychology and behavior of entrepreneurs more effectively (Masuda et al., 2018). In contrast, other studies maintain a negative relationship, describing a large firm's failure to continue entrepreneurship in the emerging market despite having sufficient and high entrepreneurial passion (Yang et al., 2021). This ambiguous relationship between entrepreneurial passion and entrepreneurial resilience suggests the existence of a moderator.

AS for the concept content of the term “Golden-mean thinking,” it was first put forward by Confucius, and is regarded as a simple philosophy of life in the Analects of Confucius representative statements such as “Too much is not enough,” “Happy but not lustful, sad but not hurt,” and so on, later Zheng Xuan, Kong Ying da, Zhu Xi and so on developed the doctrine of the golden mean into a speculative doctrine of the golden mean. However, the connotation of the doctrine of the mean has been gradually vulgarized in the long development of the world. Modern people think that “The mean” is “Eclecticism” and “Incomplete Doctrine,” and “Yong” is the representative of “Mediocrity,” including “Negative achievements, negative circumvention” of the mean is “And chaos,” “And the matter for the men” pronoun. The reason of misunderstanding lies in that the classical literature has not defined the “Golden mean” directly, only thinks that its primary connotation is “Impartial,” “Holding both sides but allowing the middle,” specific meaning is required in line the Tao of their own experience. Fortunately, since the 1980s, some Asian scholars have been studying the concept of the “Golden mean” qualitatively from the perspective of social psychology. At present, there are mainly three views on the concept connotation of the term: first, the golden mean is a kind of dialectical thinking. The doctrine of the mean is a Chinese people used to a way of thinking that all things have moderate rationality, thinking of things including dialectics and integrity. Second, the doctrine of the mean is a kind of rationality, that the doctrine of the mean is too much, the right state or to achieve such a state of a trend of action, similar to the definition of rationality, manifested in the existing principles, choose the good and stick to it. Third, moderation is wisdom. The six dimensions of intelligence are compromise, acceptance of knowledge limits, flexibility/flexibility, perspective substitution, acceptance of change, and provision of conflict resolution. Many authors hold that mean thinking mainly refers to the metacognition that an individual can consider things from many angles and take a behavior model that can take into account the overall situation and coordinate contradictions after weighing the views and information of all sides (Yang et al., 2021). Chen and Ling (2010) found that the golden mean thinking concerns the relationship between the whole and its components, looking at problems from a pluralistic perspective, establishing connections and integrating different points of view, at the same time, understanding the unity and struggle of contradictions, and pursuing the harmonious development of things, it embodies individual action goal and value, and divided it into three dimensions: pluralism, integration and harmony. Integration refers to the individual thinking about the problem when their internal ideas and the external environment are combined. Harmony is to act in a non-radical, positive, and harmonious as the principle. Drawing on the research of Chen and Ling (2010), mean thinking is defined as the thinking mode of how to integrate external conditions, and internal needs and take practical actions in a particular situation, which includes three dimensions: multi-thinking, integration and harmony.

AS for the method of measurement of the term “Golden-mean thinking,” there are several representative statements on the dimensionality of this concept at present, and the representative dimensionality is summarized in Table 1, as shown in Table 1. Given the wide application and applicability, this research chooses the three-dimensional partition to design the questionnaire for this concept.

**Table 1:** The representative scale of mean thinking.

**Year**	**Researchers**	**Dimensions**
1997	Yang Zhong Fang and Zhao Zhi Yu	Eight dimensions: the overall situation for the heavy, do not go to extremes, heaven and man in one, retreat to advance, reasonable, consider the consequences, handle contingency, wait and see the situation
2000	Zhao Zhi Yu	Three dimensions: neutralization, overall view of the overall situation, implement
2010	Chen and Ling	Three dimensions: multi-thinking, integration, harmony
2012	Yang Zhong fang and Lin Sheng Dong	Four dimensions: Philosophy of life, event handling, reflection after the event, mental health
2014	Lee Kai-Ming and Chan Chi-ha	Three dimensions: self, interpersonal, work

Based on previous qualitative research on the doctrine of the mean, more and more scholars have carried out relevant empirical research. Chen and Ling (2010) believes that the golden mean affects the psychological state and continuous willingness of entrepreneurs and that the high-golden mean entrepreneurs experience relatively little lousy mood, the same time better at psychological adjustment and state recovery. The entrepreneurs with higher golden-mean thinking also have environmental solid control and increased life satisfaction, can show different self-roles according to the situation and deliver higher self-confidence and entrepreneurial continuity. A few researchers also looked at young people about to enter society and conducted a series of studies on college students. The results showed that self-awareness, emotional intelligence, and so on were all positively affected by moderation. The college students with a high golden-mean thinking also show high healthy competitive attitude, they can deal with the competitive problems in a reasonable way, and thus have a positive predictive effect on the entrepreneurial will (Chen and Ling, 2010). Golden-mean thinking also affects the way of thinking and behavior of employees, reflected in the performance of differentiated behavior (Bing and Dong, 2015). The existing literature mainly includes the research results of the golden mean thinking on employees' innovative behavior, forward-looking behavior, advice behavior, and so on. Employees with a more moderate mindset are considered to be good at thinking from multiple perspectives, collecting and integrating different knowledge information, and adopting appropriate approaches based on organizational harmony. Therefore, most researchers believe that the golden mean thinking has a positive impact on employees' forward-looking behavior, advice behavior, learning behavior and so on, and has passed the empirical test (Wang et al., 2022). However, some scholars put forward that mean thinking has an inverted U-shaped effect on employees' creative behavior, and moderate mean thinking has a positive predictive effect on innovative behavior. Still, excessive mean thinking is easy to makes the staff “Timid,” but not conducive to innovation activities.

There have been many attempts to confirm the existence of a moderator in fostering and upgrading entrepreneurial resilience (Zahra et al., 2019). The relationship between entrepreneurial resilience and its determinants or outcomes can be altered not only by endogenous factors, such as personal ability but also by exogenous elements, including the cultural environment in which entrepreneurs grow up (Zhou and Li, 2022). Many studies have shown that thinking modes determine behavior patterns. However, few studies have investigated the impact of the golden mean thinking mode of Confucian traditional culture despite it being a good predictor of future behavior. The effect of entrepreneurial passion on entrepreneurial resilience is expected to be of different strengths depending on Confucian traditional golden-mean thinking.

Confucian traditional golden-mean thinking is a very distinct and crucial philosophical thought in Chinese traditional culture (Zeng and Wang, 2017). It is not only an ethical and moral concept in Chinese history but also a mode of thinking. In applying the golden mean, an individual will constantly monitor changes in the environment, keep an eye on whether his actions deviate from the goal of “harmony,” adjust his actions through self-reflection, and take into account a variety of positions and different points of view in resolving disputes, adopt a multi-faceted approach and make concessions easily (Huang, 2017). In other words, in the operation of the golden mean thinking, individuals must not only be aware of the inner self and adjust to the external self-behavior but also be based on the external environment and change (Li, 1998). Therefore, the doctrine of the mean not only implies personal awareness of the internal self, but also includes the adjustment and awareness of the external self-expression in different environments. Confucian traditional golden-mean thinking has rarely been investigated as a moderator in fostering and upgrading entrepreneurial resilience, even though it is one of the most established and researched variables in entrepreneurship literature.

Based on this logic, this study assumes that the strength of entrepreneurial passion could be altered by Confucian traditional golden-mean thinking (Lin, 2021). We regard Confucian traditional golden-mean thinking as a moderator at the personal level. This view is aligned with social network theory, whereby the influence of social networking on business performance is moderated by cultural factors (Zhang and Tsang, 2021). Therefore, we predict that there will be a strong relationship between entrepreneurial passion and entrepreneurial resilience when Confucian traditional golden-mean thinking is high, leading to the following hypothesis:

**Hypothesis 5a:** Confucian traditional golden-mean thinking moderates the relationship between entrepreneurial passion and entrepreneurial resilience. This positive relationship is much stronger for those with a high degree of Confucian traditional golden-mean thinking.

Assuming that Confucian traditional golden-mean thinking moderates the relationship between entrepreneurial passion and entrepreneurial resilience, it is also plausible that an entrepreneur's characteristics might conditionally affect the strength of the indirect relationship between social capital and entrepreneurial resilience (Guiso et al., 2020). In other words, the effect of passion gained from trustworthy networks on entrepreneurial resilience (mediation effect) may be moderated by Confucian traditional golden-mean thinking, thereby demonstrating a moderated mediation effect. As we assume a strong association between entrepreneurial passion and entrepreneurial resilience when Confucian traditional golden-mean thinking is high, we expect that Confucian traditional golden-mean thinking will positively moderate the mediation effect (Ellison et al., 2020). That is, the mediation effect will is stronger when Confucian traditional golden-mean thinking is high, as claimed in the following hypothesis:

**Hypothesis 5b:** Confucian traditional golden-mean thinking moderates the indirect effect of social capital on entrepreneurial resilience (*via* harmonious entrepreneurial passion and obsessive entrepreneurial passion, respectively). Specifically, entrepreneurial passion positively mediates the indirect effect when Confucian traditional golden-mean thinking is high.

**Figure 1:**
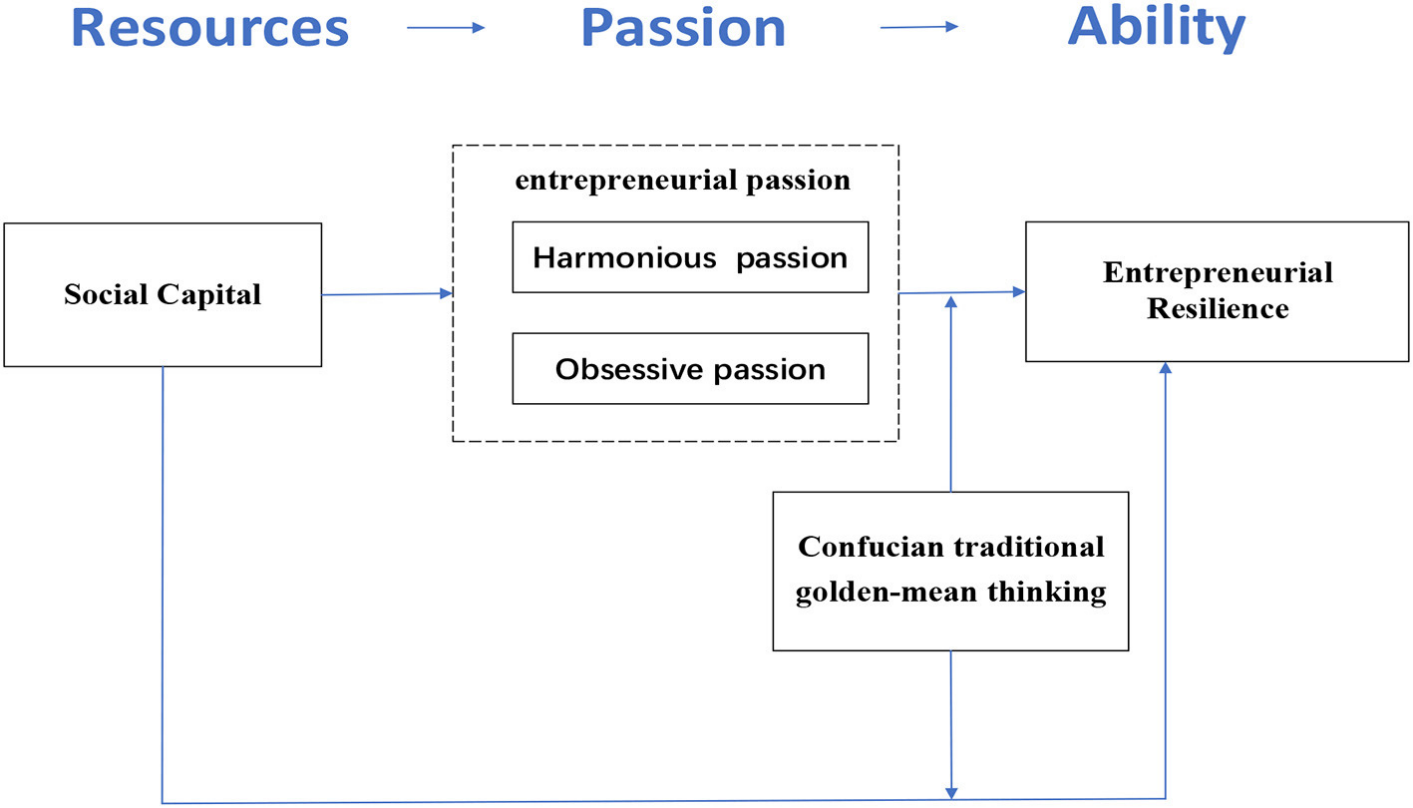
The research model.

Based on the above-proposed hypotheses and the theoretical foundation, the conceptual association among variables is presented below in Figure 1.

## Materials and methods

### Sample selection and data collection

The study defines SMEs as new start-ups whose number of employees is <250 (Dai et al., 2022). An SME entrepreneur is defined as the founder of a new start-up with <250 employees in China. Therefore, data were collected from entrepreneurs located in China who had started their own business <5 years ago and with <250 employees (Sobczak and Głuszczuk, 2022). Considering the data availability, one thousand questionnaires were distributed by telephone calls and Tencent meetings, and 690 responses were collected. After excluding missing data and outliers based on boxplot analyses, 525 responses were analyzed. To ensure the quality of the research results and a smooth process, the research group communicated with each other over the phone to indicate the intention of the research. We contacted the entrepreneurs and presented our research purpose for obtaining permission to conduct the study (Jaskiewicz et al., 2015). Then make an appointment with the founders to fill in the questionnaire. Due to the epidemic, telephone calls, Tencent meetings, and other methods were selected for full communication so that respondents could fill in the questionnaire more conveniently (Santoro et al., 2020). This study used two ways to reduce the common method bias. ① During transmission, the respondents were told that the research data were only for academic research, and there was no difference between right and wrong in all answers. They also promised to keep the obtained data strictly confidential, dispel the psychological concerns of the subjects, and do an excellent job of psychological isolation, so that the respondents could answer the questions more accurately. ② Collect data in two-time points (3–4 weeks). Each questionnaire is divided into parts A and B. Each start-up was numbered, and the questionnaires collected by the same enterprise were matched and merged. The survey was conducted from September 2021 to February 2022.

In order to ensure the representativeness of the research sample, we made efforts to expand the distribution of questionnaires and enrich the data collection methods. We distributed questionnaires within China, rather than in one or several specific provinces, which increased the sample distribution breadth, avoided the difference bias to some extent, and improved the sample representativeness.

We calculated the following statistics based on demographic data. The results of descriptive statistical analysis of sample enterprises are shown in Table 1. As shown in Table 1, 240 respondents were in their 30 s (43.0%), 218 respondents were in their 40 s (39.1%), 67 respondents were in their 50 s (12.0%), and 33 respondents were in their 60 s (5.9%). Among all respondents, 196 had a bachelor's degree or higher (35.1%). “Electric, electronics, communication and precision” was the most popular industrial category, accounting for 47.7% of all respondents. Regarding their work of experiences, 40.9% of all respondents had 1–3 years experience working within the same industry. Table 1 presents the demographic information of the research sample.

### Variable measurement

Taking into consideration the focus of this study, we included only entrepreneurs in our sample. The entrepreneurs who owned entrepreneurial start-ups with <250 employees in China were selected using a non-probability (convenience) sampling method. The sample size is 525, as recommended by Sobczak and Głuszczuk (2022). All items were presented on a 7-point scale, from 1 (strongly disagree) to 7 (strongly agree).

The measurement of entrepreneurs' social capital was based on the rationale proposed by Yao and Meng (2022). Respondents were asked to rate how many social network resources the entrepreneurs have which can be translated into entrepreneurial activities. Items included “You have established a good relationship with the residents of the place where Your Business is located” and “Your relatives or friends give you advice or financial support.” For this construct, Cronbach's alpha was 0.936. According to Nunnally (1978), reliability of 0.70 or better is recommended (Nunnally, 1978). Hence, this value has sound scale reliability.

The entrepreneurial Passion scale developed by Vallerand et al. was used to measure the degree of positive emotional experience in the entrepreneurial process. It has two subscales: harmonious entrepreneurial passion and obsessive entrepreneurial passion. As for harmonious entrepreneurial passion, Sample items included “Entrepreneurship has enriched my life experience, in the process of entrepreneurship, I found many very meaningful new things,” “Entrepreneurship has brought me many very memorable experiences, entrepreneurial success requires personal quality is what I aspire to.” For this construct, Cronbach's Alpha was 0.936. Obsessive entrepreneurial passion scale including four items, Items included “I can't live without entrepreneurship. I can't imagine life without it,” “I was emotionally dependent on the satisfaction of starting a business, and I had trouble controlling my desire to do so,” with a Cronbach's alpha of 0.938, reasonable scale reliability.

The entrepreneurial resilience scale adopted from Fatoki (2018) was modified to measure the entrepreneur's mental ability to recover and adjust after facing difficulties or adversity. Sample items included “I can overcome difficulties to achieve the goal, not easily discouraged by failure,” “I consider myself a very strong person and can stay focused under pressure.” For this construct, Cronbach's Alpha was 0.935.

In terms of the procedure of the scale, taking into account the representativeness, applicability and data availability of the scale, we mainly adopted the research of Luo et al. (2020). Their “golden-mean thinking scale” measures three levels of event-specific processing: “Multi thinking” before dealing with the problem, “Integration” when choosing a solution, and “Harmony” when implementing a solution. The scale is suitable for people to deal with different opinions in conflict situations and has more pertinence in corporate events. It has been adopted by many relevant researchers in organizational behavior. Sample items included “When discussing opinions, I take into account the conflicting opinions and consider all possible situations,” “I try to find a balance between my own opinions and those of others. I will adjust my original idea after considering others' opinions,” “When I make a decision, I usually consider the harmony of the overall atmosphere and usually adjust the way I express myself for the sake of overall harmony,” “I have been deeply influenced by the traditional culture of Confucianism since I was a child.” For this construct, Cronbach's Alpha was 0.935.

Control variables. The control variables in this study include the establishment years, annual turnover, and yearly innovation investment. These variables were controlled for since similar establishment years, annual turnover, and annual innovation investment would be helpful toward the entrepreneurial resilience.

## Empirical analysis and results

To test the hypotheses, this study has used a mediated model with regulation. It is a statistical method that comprises mathematical and statistical approaches for examining data to identify relationships between variables. Employing the software SPSS and PROCESS, this study has conducted SPSS and PROCESS3.3. These methods are helpful in measuring mediating and moderating effects and are suitable for the exploratory nature of study analysis. In recent years the number of published articles using mediated models with regulation increased significantly.

### Reliability analysis

It is essential to check the reliability and validity of measurement tools (Yang et al., 2016). Reliability analysis verifies the internal consistency of the scale, that is, whether different items can measure the same content or concept independently. Cronbach's Alpha coefficient is mainly used in this paper to investigate the internal consistency of the scale. Cronbach's Alpha coefficient is between 0 and 1. If the α coefficient does not exceed 0.6, the internal reliability is generally considered inadequate. When the α coefficient reaches 0.7–0.8, the scale has considerable reliability. When the α coefficient comes 0.8–0.9, the reliability of the scale is excellent. As shown in Table 2, the total scale's reliability of this study is 0.984, more significant than 0.9. Cronbach's Alpha of all dimensions of the scale is more significant than 0.9. The results show that the scale and dimensions have high reliability, good stability and consistency and can be used for in-depth analysis.

**Table 2:** Demographics of survey respondents.

**Variable**	**Category**	***N***	**Percentage (%)**
Gender	Male	283	50.7
	Female	275	49.3
Age	The 20s	0	0.0
	The 30s	240	43.0
	The 40s	218	39.1
	The 50s	67	12.0
	The 60s	33	5.9
Education	Junior college and below	20	3.6
	Bachelor's degree	196	35.1
	Master's degree	235	42.1
	Doctoral degree	107	19.2
Industry type	Non-metal, metals, machine equipment	163	29.2
	Computer and office machine	87	15.6
	Electric, electronics, communication and precision	266	47.7
	Daily supplies	35	6.3
	Other	7	1.3
Work experience	1–3	228	40.9
in the same industry	4–5	160	28.7
	6–8	139	24.9
	9	31	5.6

### Validity analysis

Validity refers to the validity of the investigation methods, means and results. In other words, validity refers to the accuracy of the survey method and the data obtained, that is, accuracy. The commonly used validity test methods mainly include content validity and construct validity (Breugst et al., 2012). Among them, content validity refers to whether the design items of the questionnaire determine the subject to be studied. Because this paper is based on the reference of existing domestic and foreign literature, there are relevant references for the grade seven scale of each research variable. After completing the questionnaire design, the author referred to the opinions of the instructor and classmates. Then the expression of the question and the structure of the questionnaire were modified to form the Firstly draft of the questionnaire. Before the formal investigation, the questionnaire was tested in a small range, and ambiguous and repetitive questions were modified and deleted. Therefore, it can be concluded that the questionnaire has good content validity. The consistency between structure and theoretical structure reflected by measurement results is structure validity. The structural validity reflects whether the experiment really measures the hypothesis theory. Exploratory factors will be used to measure the scale's structural validity. The purpose of exploratory factor analysis is to identify several common factors that can represent the basic framework of the scale. The degree of correlation between observed variables and common factors was also reflected.

Data should pass the KMO sample measure and Bartlett's test of Sphericity before performing the corresponding factor analysis (Chen et al., 2019). To verify the partial correlation and simple correlation coefficient between variable items. When the correlation is high, the data is suitable for factor analysis. Kiser gives common a KMO metrics: the KMO value above 0.9 indicates that it is very suitable for factor analysis. 0.8–0.9 is suitable; 0.7–0.8 is fair; 0.6–0.7 is acceptable; 0.5–0.6 indicates not suitable. Less than 0.5 is very unsuitable. As can be seen from the above table, the KMO value is 0.972, >0.9, indicating that the data is suitable for factor analysis. Bartlett's chi-square value of the sphericity test is 15,875.613 (P <0.01), indicating that the relationship between various items of user variables is good enough for factor analysis. Interpret the eigenvalues of the full variance observation scale, extract the sum of the squares of loads. And then we rotate the sum of the squares of the load and the cumulative percentage. In this paper, the cumulative percentage is mainly observed. If it exceeds 50%, it meets the requirement of factor analysis.

Following the analytical procedure suggested by Hair et al. (2010), confirmatory factor analysis is used to validate the construct measures. The findings in Table 2 indicate the criteria for convergent validity and reliability. We then adopt a two-step analytical strategy (Hair et al., 2010) through which we confirm the measurement model by using confirmatory factor analysis and using a structural equation model to estimate the fit of the structural model to the data. To mitigate and assess the magnitude of standard method variance, we adopt the procedural remedies and statistical methods suggested by Podsakoff et al. (2003). Respondents were assured of anonymity and confidentiality to reduce evaluation apprehension. Harman's one-factor test was conducted, extracting nine distinct factors that accounted for 75% of the total variance, with the first factor explaining 25%. Results demonstrate that no single factor emerges, nor does one-factor account for most of the variance, indicating the little possibility of standard method variance. A total of 5 factors were extracted to explain 93.467% of the total variance. The value is >50%, indicating that the extracted five factors can well explain the information contained in the original variables. Item factor load of each dimension is >0.5. And each topic is within the dimensions originally defined, without variable confusion. This shows that the model has high structural validity.

### Correlation analysis

Table 3 shows the descriptive statistics and correlations for the variables included in the study. In the correlation analysis of various numerical variables, the commonly used statistical analysis method is the Pearson correlation coefficient (Ensley, 1997). Academics use it to measure correlations between things or variables. The academic circle reveals and reflects the correlation between different things or variables through numerical quantification (Omri and Boujelbene, 2015). As can be seen from Table 3, the mean values of social capital, harmonious entrepreneurial passion, obsessive entrepreneurial passion, and entrepreneurial resilience are 4.732, 5.487, 5.637, and 5.341, respectively. These values are in the middle. It indicates that the social capital, the level of entrepreneurial passion, and entrepreneurial resilience of entrepreneurial need to be improved. The average level of Confucian traditional golden-mean thinking is 5.376, indicating that Confucian traditional golden-mean thinking is at a high level. All the variables showed a positive correlation. As shown in Table 3. This provides preliminary support for the above research hypothesis.

**Table 3:** Results of confirmatory factor analysis (*N* = 525).

**Variable**	**Items**	**α coefficient**	**Standardized factor loading**	**Average variance extracted (AVE)**	**Composite reliability (CR)**
X social capital	X1 You have established a good relationship with the residents of the place where Your Business is located	0.939	0.852	0.769	0.936
	X2 Your relatives or friends give you advice or financial support	0.940	0.882		
	X3 You have a good relationship with customers and suppliers, often participate in a variety of business groups activities	0.938	0.848		
	X4 You have a good relationship with the government departments involved in business affairs	0.937	0.704		
	X5 You have good cooperation with local universities and scientific research institutions	0.937	0.580		
M1 harmonious entrepreneurial passion	M1–1 Entrepreneurship has enriched my life experience. In the process of entrepreneurship, I found many very meaningful new things	0.938	0.731	0.706	0.936
	M1–2 Entrepreneurship has brought me many very memorable experiences, entrepreneurial success requires personal quality is what I aspire to	0.938	0.812		
	M1–3 Entrepreneurship is in harmony with the rest of my life. I'm fascinated by entrepreneurship. I love it	0.937	0.696		
M2 obsessive entrepreneurial	M2–1 I can't live without entrepreneurship. I can't imagine life without it	0.939	0.802	0.658	0.938
passion	M2–2 I was emotionally dependent on the satisfaction of starting a business, and I had trouble controlling my desire to do so	0.939	0.769		
	M2–3 For Entrepreneurship, I have a compulsive feeling, my mood is greatly affected by entrepreneurship	0.939	0.742		
	M2–4 I have a strong inner urge to start a business, I can't help but want to start a business	0.938	0.936		
W confucian traditional	W1 When discussing opinions, I take into account the conflicting opinions and consider all possible situations	0.936	0.738	0.617	0.935
golden-mean thinking	W2 I try to find a balance between my own opinions and those of others and adjust my original idea after considering others' opinions	0.936	0.781		
	W3 When I make a decision, I usually consider the harmony of the overall atmosphere and usually adjust the way I express myself for the sake of overall harmony	0.936	0.789		
	W4I have been deeply influenced by the traditional culture of Confucianism since I was a child	0.935	0.882		
Y entrepreneurial	Y1 I can overcome difficulties to achieve the goal, not easily discouraged by failure	0.938	0.676	0.608	0.935
resilience	Y2 I consider myself a very strong person and can stay focused under pressure	0.937	0.792		
	Y3 It's easy for me to get back to good form as soon as possible after a difficult situation and be able to cope with any difficulties I encounter	0.937	0.755		
	Y4 I can adapt to change, cope with the pressure makes me strong	0.936	0.649		
	Y5 I try to look on the bright side of things and be able to withstand unpleasant feelings	0.937	0.592		

N = 525; independent variable X stands for social capital; dependent variable Y stands for entrepreneurial resilience; mediator variable M1 stands for Harmonious Entrepreneurial Passion; mediator Variable M2 stands for Obsessive Entrepreneurial Passion; mediator variable W stands for Confucian traditional golden-mean thinking.

### Mediation effect of entrepreneurial passion

We adopted Baron and Kenny's suggestion to test the mediation effect (H1, H2, H3, and H4) of entrepreneurial passion (harmonious entrepreneurial passionand obsessive entrepreneurial passion) between social capital and entrepreneurial resilience. According to Baron and Kenny, four requirements should be met to assess mediation effect. First, the independent variable X and the mediation variable M (M1 and M2) should each be regressed on the dependent variable Y. The variable X should also be regressed on the variable M. Partial mediation effect is confirmed if the variable X remains important and its effect becomes smaller while controlling the variable M. Full mediation effect occurs if the variable X is no longer significant.

The results show that social capital is positively related to entrepreneurial resilience (β = 0.2578, *t* =10.1427, *p* < 0.001) and harmonious entrepreneurial passion (β = 0.2642, *t* = 12.7292, *p* < 0.001), respectively. harmonious entrepreneurial passion is also positively associated with entrepreneurial resilience (β = 0.3022, *t* = 8.8762, *p* < 0.001). At the same time, social capital is positively related to obsessive entrepreneurial passion (β = 0.0778, *t* = 2.3017, *p* < 0.001), obsessive entrepreneurial passion is also positively associated with entrepreneurial resilience (β = 0.2205, *t* = 6.2191, *p* < 0.001). Thus, H1, H2, and H3 are statistically supported.

The effect of social capital on entrepreneurial resilience was 0.355 (*p* < 0.001) while not controlling the variable M (entrepreneurial passion). The effect of social capital on entrepreneurial resilience was 0.2578, while controlling the variable M (entrepreneurial passion), confidence interval [0.2079, 0.3078], excluding 0, the test result was significant. The effect of social capital on entrepreneurial resilience decreased from 0.355 to 0.2578, while controlling the variable M, indicating that entrepreneurial passion played an intermediary role. The effect of social capital is still significant, but the size of its effect is diminished when controlling for entrepreneurial passion. Hence, the partial mediation effect of entrepreneurial passion is confirmed.

**Figure 2:**
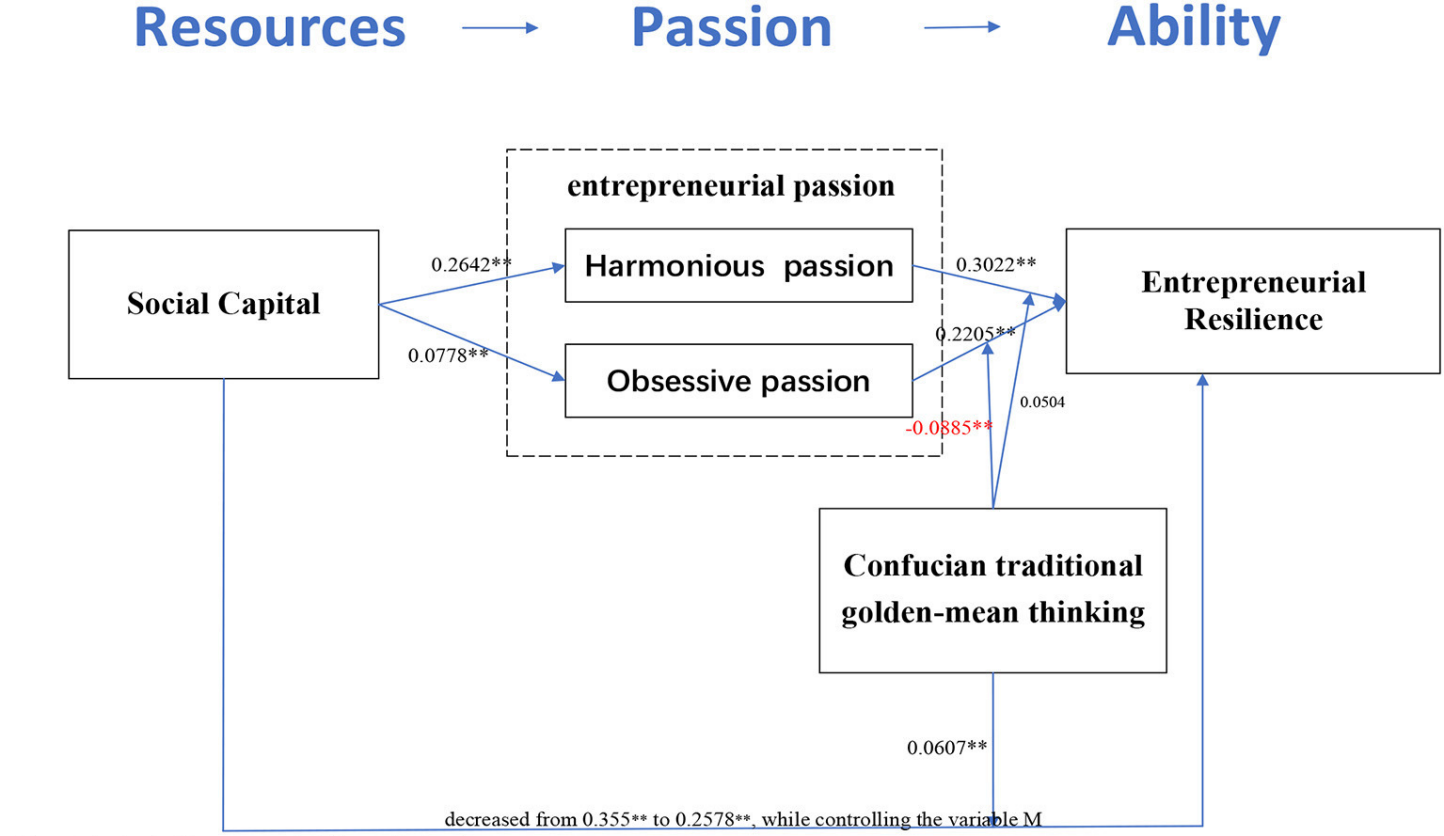
Summary of coefficients inthe integrated model. ^*^Correlation is significant at *P* < 0.05 (two-tailed test). ^**^Correlation is significant at *P* < 0.01 (two-tailed test).

Moreover, the Bootstrap method was then used to examine the mediating effect of the model further. Set Bootstrap to 1,000 cycles with 95% confidence interval. The result of the bootstrapping analysis also shows that the indirect effect of social capital on entrepreneurial resilience is statistically significant [① as for harmonious entrepreneurial passion, indirect effect=0.113, 95% bias-corrected CI (0.071, 0.160); ② as for obsessive entrepreneurial passion, indirect effect=0.024, 95%bias-corrected CI (0.003, 0.049)]. As a result, entrepreneurial passion partially mediates the relationship between social capital and Entrepreneurial resilience, meaning H4 is supported. The bootstrapping results of these four hypotheses are presented in Figure 2.

### Moderating effect of Confucian traditional golden-mean thinking

**Figure 3:**
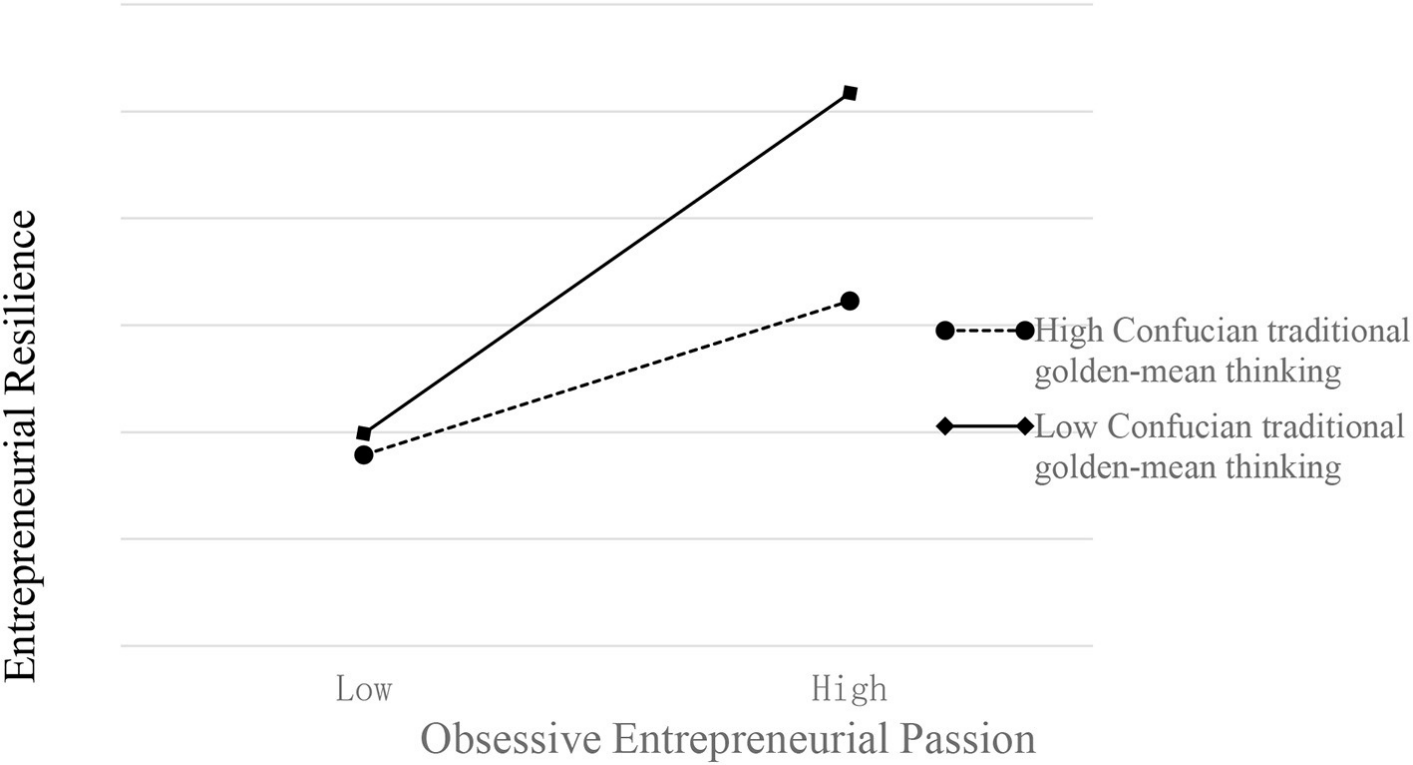
Cross-level moderating effects of Confucian traditional golden-mean thinking.

As Table 4 showed, H5a postulates that the effect of social capital on entrepreneurial resilience would be positive for entrepreneurs with high Confucian traditional golden-mean thinking. This hypothesis was tested using hierarchical regression analysis. The result shows that the model's explanatory power increases when the interaction term is included in the regression equation, as Table 4 showed (β = 0.0607, *t* = 2.5415, *p* < 0.001). The main effects of harmonious entrepreneurial passion (β = 0.2480, *t* = 7.3080, *p* < 0.001), obsessive entrepreneurial passion (β = 0.1151, *t* = 3.1373, *p* < 0.001), and Confucian traditional golden-mean thinking (β = 0.2859, *t* = 7.4296, *p* < 0.001) on entrepreneurial resilience are also significant. Each of the variable's variance inflation factors (VIF) is <1.00. Table 4 reports the results of the moderation effect. We also conducted a slope test to identify the pattern of this moderation effect, as suggested by Aiken and West (1991). Figure 3 presents the result of the slope test.

**Table 4:** Descriptive statistics and correlations of the variables.

**Variable**	**Mean**	**SD**	**X**	**M1**	**M2**	**W**	**Y**
X	4.732	1.151					
M1	5.487	0.909	0.305**				
M2	5.637	0.825	0.110*	0.490**			
W	5.376	0.896	0.489**	0.508**	0.434**		
Y	5.341	0.816	0.499**	0.581**	0.444**	0.618**	1

* Correlation is significant at p < 0.05 (two-tailed test).

** Correlation is significant at p < 0.01 (two-tailed test).

N = 525; independent variable X stands for social capital; dependent variable Y stands for entrepreneurial resilience; mediator variable M1 stands for Harmonious Entrepreneurial Passion; mediator Variable M2 stands for Obsessive Entrepreneurial Passion; mediator variable W stands for Confucian traditional golden-mean thinking.

In contrast with our expectation, the result indicates that an entrepreneur's obsessive entrepreneurial passion has a more substantial impact on entrepreneurial resilience when Confucian traditional golden-mean thinking is low (β = −0.0885, *t* = −2.6789, *p* s< 0.001). On the other hand, the slope is relatively weak for those with high Confucian traditional golden-mean thinking. Hence, the interaction effect between obsessive entrepreneurial passion and Confucian traditional golden-mean thinking is statistically confirmed. Note, however, that its direction is opposite to what was assumed in H5a. This is a very significant finding.

As for the harmonious entrepreneurial passion, although the Confucian traditional golden-mean thinking has been proved to have a positive moderating effect on the positive relationship between harmonious entrepreneurial passion and entrepreneurial resilience, the moderating effect is not significant [β = 0.0504, *t* = −1.6893, *p* < 0.05, 95%bias-corrected CI (−0.0082, 0.1091), including 0].

Having confirmed that the moderation effect is supported, we further analyzed the moderated mediation effect (H5b). SPSS PROCESS was used to compute the moderated mediation effect at various values of the moderator. As Table 5 showed, the output of this analysis provides detailed results of the interaction effect by presenting its statistical significance at the degree of Confucian traditional golden-mean thinking one standard deviation above and below the mean.

**Table 5:** Hierarchical regression analysis of moderation effect (*N* = 525).

**Action path**	**Model 1: Mediation model**				**Model 2: Moderated mediation model**				
	**Coefficients**	**Standard error**	***T*-value**	**95% confidence interval**	**Coefficients**	**Standard error**	***T*-value**	**95% confidence interval**	
				**(Upper, Lower)**				**Upper**	**Lower**
X → M1	0.2642**	0.0352	12.7292	(0.1950, 0.3350)	0.2642**	0.0352	7.4996	(0.1950,0.3335)	
X → M2	0.0778**	0.0338	2.3017	(0.0114, 0.1443)	0.0778**	0.0338	2.3017	(0.0114, 0.1443)	
M1 → Y	0.3022**	0.0340	8.8762	(0.2353, 0.3690)	0.2480*	0.0339	7.3080	(0.1813, 0.3146)	
M2 → Y	0.2205**	0.3550	6.2191	(0.1509, 0.2902)	0.1151**	0.0367	3.1373	(0.0430, 0.1871)	
X → Y	0.2578**	0.0254	10.1427	(0.2079, 0.3078)	0.1801**	0.0266	6.7738	(0.1279, 0.2323)	
W → Y					0.2859*	0.0385	7.4296	(0.2103, 0.3615)	
X*W → Y					0.0607**	0.0239	2.5415	(0.0138, 0.1076)	
M1*W → Y					0.0504	0.0298	1.6893	(−0.0082, 0.1091)	
M2*W → Y					−0.0885**	0.0331	2.6789	(−0.1535, −0.0236)	
K1 → Y	0.1274*	0.0525	2.4252	(0.0242, 0.2306)	0.0877	0.0501	1.7493	(−0.0108, 0.1862)	
K2 → Y	0.0410	0.0328	1.2498	(−0.0234, 0.1054)	0.0442	0.0312	1.4153	(−0.0171, 0.1054)	
K3 → Y	0.0612*	0.0302	2.0240	(0.0018, 0.1205)	0.0464	0.0289	1.6061	(−0.0103, 0.1031)	
K4 → Y	0.0075	0.0263	0.2852	(−0.0441, 0.0591)	0.0069	0.0249	0.2753	(−0.0421, 0.0559)	
K5 → Y	0.0067	0.0316	0.2115	(−0.0555, 0.0688)	0.0040	0.0301	−0.1314	(−0.0630, 0.0551)	
K6 → Y	−0.0721*	0.0282	−2.5555	(−0.1276, −0.0167)	0.0576	0.0269*	−2.1407	(−0.1104,−0.0047)	
K7 → Y	0.0476	0.0560	0.8493	(−0.0625, 0.1576)	0.0457	0.0533	0.8577	(−0.0590, 0.1505)	
K8 → Y	−0.0089	0.0316	−0.2819	(−0.0710, 0.0532)	0.0033	0.0301	0.1112	(−0.0557, 0.0624)	

N = 525; independent variable X stands for social capital; dependent variable Y stands for entrepreneurial resilience; mediator variable M1 stands for Harmonious Entrepreneurial Passion; mediator Variable M2 stands for Obsessive Entrepreneurial Passion; mediator variable W stands for Confucian traditional golden-mean thinking.

** *P* < 0.01;

* *P* < 0.05.

This allows us to verify the value of Confucian traditional golden-mean thinking for which the conditional indirect effect is significant. As Table 6 showed the result demonstrates that the moderated mediation effect of obsessive entrepreneurial passion is negative, and has a non-zero probability [β = −0.0069, 95% bias-corrected CI (−0.0185, −0.0001), excluding 0]. At the same time, the moderated mediation effect of obsessive entrepreneurial passion is not significant [β = 0.133, 95% bias-corrected CI (−0.0095, 0.0403), including 0].

**Table 6:** Direct, indirect, and conditional indirect effects of X on Y.

**Mediation model**	**Direct effect (X**→**Y)**					**Indirect effect (X**→**M1**→**Y)**				
	**Coefficients**	**Standard error**	***T*-value**	**Lower**	**Upper**	**Coefficients**		**Standard error**	**Lower**	**Upper**
	0.3548	0.0287	12.3818	0.2985	0.1111	0.1127		0.0227	0.071	0.1601
						**Indirect effect (X**→**M2**→**Y)**				
						**Coefficients**		**Standard error**	**Lower**	**Upper**
						0.0242		0.012	0.0029	0.0493
**Moderated mediation model**	**Direct effect (X**→**Y)**					**Conditional indirect effect (X**→**M1**→**Y)**				
	**Coefficients**	**Standard error**	***t***	**Lower**	**Upper**		**Coefficients**	**Standard error**	**Lower**	**Upper**
	0.2859	0.0385	7.4269	0.1279	0.2323		0.133	0.0127	−0.0095	0.0403
						**Exponential grouping**	**Coefficients**	**Standard error**	**Lower**	**Upper**
						−0.8960	0.0536	0.0162	0.0239	0.0884
						0.0000	0.0655	0.0155	0.0389	0.0990
						0.8960	0.0775	0.0219	0.0410	0.1258
						**Conditional indirect effect X**→**M2**→**Y**				
							**Coefficients**	**Standard error**	**Lower**	**Upper**
							−0.0069	0.0048	−0.0185	−0.0001
						**Exponential grouping**	**Coefficients**	**Standard error**	**Lower**	**Upper**
						−0.8960	0.0151	0.0084	0.0015	0.0336
						0.0000	0.0090	0.0055	0.0005	0.0216
						0.8960	0.0028	0.0052	0.0077	0.0140

N = 525; independent variable X stands for social capital; dependent variable Y stands for entrepreneurial resilience; mediator variable M1 stands for Harmonious Entrepreneurial Passion; mediator Variable M2 stands for Obsessive Entrepreneurial Passion; mediator variable W stands for Confucian traditional golden-mean thinking.

Similar to H5a, H5b is partially supported because the moderated mediation effect is statistically significant.

## Discussion

The purpose of this study is to verify the mediation effect of entrepreneurial passion between the entrepreneur's social capital and entrepreneurial resilience and the moderation effect of Confucian traditional golden-mean thinking.

① Both dimensions of entrepreneurial passion mediate between social capital and entrepreneurial resilience. However, there are still slight differences in the influence intensity. In the process of entrepreneurial activities of Chinese SME entrepreneurs, the direct effective value of social capital on entrepreneurial resilience is 0.355, and the effect value decreases from 0.355 to 0.258 after the addition of mediating variables. The weaker effect is that entrepreneurial passion partially mediates the influence between social capital and entrepreneurial resilience. Taking a closer look, the mediating effect intensity of harmonious entrepreneurial passion is 0.0798 (0.2642^*^0.3022), and the mediating effect intensity of compulsive entrepreneurial passion is 0.0172 (0.0778^*^0.2205). It can be seen that although both dimensions of entrepreneurial passion play a mediating role in the relationship between social capital and entrepreneurial resilience, there are still slight differences in the influence intensity. Harmonious entrepreneurial passion plays a more significant and stronger mediating effect than obsessive entrepreneurial passion.

② Confucian traditional golden-mean thinking plays a positive moderating role between social capital and entrepreneurial resilience. The positive effect of social capital on entrepreneurial resilience is positively moderated by Confucian traditional golden-mean thinking. The positive adjustment intensity is 0.0607, which means that with the increase of the intensity of Confucian traditional golden-mean thinking, the direct positive effect of social capital on entrepreneurial resilience will also increase by 0.0607, which will bring beneficial enlightenment to the practice of entrepreneurs. When entrepreneurs have enough social capital, how to transform social capital into entrepreneurial resilience more effectively can be realized by improving the intensity of their own Confucian traditional golden-mean thinking. The possible reasons are as follows: On the one hand, Confucian traditional golden-mean thinking reflects the neutral action goal of being impartial and considering the overall situation; on the other hand, it demonstrates the value of pursuing the harmonious balance between the whole and the individual.

Similarly, in the face of different social relationships, entrepreneurs may have different degrees of Confucian traditional golden-mean thinking and entrepreneurial resilience. Extensive social capital itself reflects that entrepreneurs have to face a variety of connections with significant differences. In contrast, entrepreneurs with high Confucian traditional golden-mean thinking are more tolerant of such differences and willing to seek opportunities for development and progress in an environment full of contradictions. Entrepreneurial continuity and entrepreneurial resilience are more likely to emerge in this context. Entrepreneurs with a high level of moderate thinking can make more effective use of all kinds of information, and deal with contradictions and conflicts in work so that entrepreneurs have a stronger sense of control in the complex work environment. Entrepreneurs with high Confucian traditional golden-mean thinking, they are more inclined to cooperate and share helpful information and gain more innovative knowledge. In addition, entrepreneurs with a high doctrine of the Mean pursue harmony without uniformity and will find harmonious and balanced solutions when there are different views. They have better adaptability, which is also conducive to the improvement of entrepreneurial continuity and entrepreneurial resilience. However, entrepreneurs with low Confucian traditional golden-mean thinking are more constrained by existing work tasks and resources, lack tolerance for different ideas and opinions brought by external connections and social capital, cannot well adjust their ideas and thinking, and cannot reconcile their internal contradictions, are reluctant to broaden the entrepreneurial boundary, and have a high entrepreneurial failure rate and weak entrepreneurial intentions. It is not conducive to developing entrepreneurial continuity and entrepreneurial resilience. The inclusiveness and integrity reflected by Confucian traditional golden-mean thinking can help individuals adapt to complex situations, obtain adequate information and provide conditions for the generation of employees' creative ideas. Confucian traditional golden-mean thinking allows individuals to integrate external data with internal requirements and take actions on the premise of ensuring harmony, thus promoting entrepreneurship resilience of entrepreneurs.

③ Confucian traditional golden-mean thinking played a negative regulating role in the relationship between obsessive entrepreneurial passion and entrepreneurial resilience. Still, it did not regulate the relationship between harmonious entrepreneurial passion and entrepreneurial resilience. The positive effect of obsessive entrepreneurial passion on entrepreneurial resilience was negatively moderated by Confucian traditional golden-mean thinking. The adjustment intensity is −0.0885, which means that as the intensity of Confucian traditional golden-mean thinking increases, the positive effect of obsessive entrepreneurial passion on entrepreneurial resilience will decrease, hindering the improvement of entrepreneurial resilience, which will also bring beneficial enlightenment to the practice of entrepreneurs and help entrepreneurs with low Confucian traditional golden-mean thinking avoid such negative trap. When the entrepreneur has strong enough obsessive entrepreneurial passion, the thinking mode can appropriately weaken Confucian traditional golden-mean thinking. Possible reasons are as follows: Obsessive entrepreneurial passion and harmonious entrepreneurial passion inspire entrepreneurial resilience differently. Obsessive entrepreneurial passion mainly inspires entrepreneurial resilience through identity compulsion, which means that entrepreneurs must form their inherent working style and identity authority to some extent, which is precisely contrary to the “harmony” and “softness” in Confucian traditional golden-mean thinking. Therefore, when the intensity of Confucian traditional golden-mean thinking is high, the entrepreneurs will face the conflict between the “self-authority” brought by the obsessive entrepreneurial passion and the “take the overall situation” brought by Confucian traditional golden-mean thinking and will hesitate in making decisions. Hence, it is not conducive to the improvement of entrepreneurial resilience.

### Implications

The possible implications of this study are mainly reflected in three aspects:

#### Theoretical implications

Existing studies present a variety of multidimensional patterns, which provide a variety of ideas for understanding the trend of entrepreneurial resilience toward three-dimensional. These multi-dimensional research perspectives can provide broad ideas and multiple possibilities for understanding the rich features of entrepreneurial resilience. The formation and stimulation of entrepreneurial passion is regarded as an internal process and social capital are emphasized as the critical factor in cultivating entrepreneurial resilience. In addition, deep and rich social capital has a direct impact on entrepreneurial passion and an indirect effect on entrepreneurial resilience. Therefore, entrepreneurs are better at fostering and enhancing entrepreneurial resilience by stimulating and accumulating entrepreneurial passion. Starting from traditional culture, the moderating effect of the golden-mean thinking is verified, the boundary conditions are expanded, and the applicability of social capital of entrepreneurs in management practice is enhanced. Currently, most management studies ignore the local characteristics of developing countries, preferring theories deduced from the management context of developed countries, lacking local studies. Chinese traditional culture is extensive and profound and contains rich management wisdom, which is worthy of being deeply explored and integrated into modern management practice by scholars from developing countries. Based on the local context of China, this study enhances the connection between “harmony” culture and management, and further enriches the theory of social capital.

#### Method implications

This study proposes a novel and rich model, incorporating social capital, entrepreneurial passion, entrepreneurial resilience, and the golden-mean thinking into a comprehensive research framework, avoiding the fragmented research results in the current literature, and realizing the integration of resources, emotions, and culture in the process of entrepreneurship.

#### Practical implications

In recent years, there has been an increase in the research literature on the entrepreneurship policies of SMEs by governments of various countries, but the research content is relatively conservative and fuzzy, lacking policy implications and theoretical significance. Entrepreneurs should adapt to the development of the Times and understand the diversification of world development. The tolerance in the golden-mean thinking is not only an effective management wisdom. Inclusiveness is the core force for entrepreneurs to improve their cognition and entrepreneurial resilience. This study is helpful for SME entrepreneurs in China to better understand and play the essential role of diverse and highly heterogeneous social capital and effectively stimulate and enhance entrepreneurial resilience. As entrepreneurial resilience continues to play an essential component of entrepreneurial success, our findings offer substantial implications for policymakers and training programs for those with a low Confucian tradition of moderation. Based on the above research results, compared with the harmonious entrepreneurship passion, the compulsive entrepreneurship passion has a more substantial effect on the entrepreneurs with a lower level of the traditional Confucian doctrine of Mean thinking. This is important because early startup success highly depends on an individual's mindset. With a harmonious entrepreneurial passion, people with a high degree of the Traditional Confucian doctrine of the Mean can achieve entrepreneurial resilience.

In contrast, those with a low Confucian tradition of philosophy of the Mean were better able to cultivate and promote entrepreneurial resilience when they had a high degree of obsessive entrepreneurial passion. Therefore, compared with the entrepreneurship education of the high Confucian traditional doctrine of the Mean, it is beneficial and practical to encourage and accumulate the “new generation of entrepreneurs” with the low Confucian conventional doctrine of the Mean. Based on the Situation of China, entrepreneurs in small and medium-sized enterprises can cultivate their thinking of the golden-mean thinking from three aspects of multi-thinking, harmony, and integration.

### Limitations and future research directions

Inevitably, this study has some limitations: (1) With the strength of Confucian traditional golden-mean thinking as a moderating variable, this study explains the contingency effect of Confucian culture on the path of “social capital-entrepreneurial passion-entrepreneurial resilience.” However, there are still many cultural factors. More efforts should be made to explore moderators since the entrepreneurial process is highly context-dependent. The present study investigated Confucian traditional golden-mean thinking as a moderator. However, other factors, such as Taoist culture and Buddhist culture, could moderate the strength of the effects on entrepreneurial success and warrant further investigation. (2) The research object of this paper is new ventures. Social capital is a complex issue and plays a heterogeneous role in developing enterprises. In the context of Localization in China, this study can use a more dynamic perspective to track whether social capital continues to have a positive impact on corporate resilience by taking each step of enterprise development as the research object. (3) Entrepreneurial resilience is a complex issue (Kai et al., 2021). Future research can try to introduce cross-hierarchical analysis or configuration methods to integrate factors at different levels, such as environment, organization, team and employee, into the study.

## Conclusion

This study has examined social capital on entrepreneurial resilience. It has also analyzed the role of Confucian traditional golden-mean thinking in these relationships. Hypothesis 1 is accepted, showing that social capital has positively influence on entrepreneurial resilience (Hao et al., 2015). Hypothesis 2 is also taken, showing that social capital has positive influences on entrepreneurial passion. Hypothesis 3 is accepted, showing that entrepreneurial passion has positive consequences on entrepreneurial resilience. And hypothesis 4 is also accepted, confirming that entrepreneurial passion plays a mediating role between social capital and entrepreneurial resilience. While verifying the moderating effect of Confucian traditional golden-mean thinking, this study found the opposite conclusion, which would be a significant finding.

Contrary to common assumptions, Confucian traditional golden-mean thinking negatively moderates the relationship between obsessive entrepreneurial passion and entrepreneurial resilience. The positive relationship between obsessive entrepreneurial passion and entrepreneurial resilience would be much stronger for those with a low degree of Confucian traditional golden-mean thinking. This is a very significant finding. Although the Confucian traditional golden-mean thinking has been proved to have a positive moderating effect on the positive relationship between harmonious entrepreneurial passion and entrepreneurial resilience, the moderating effect is not significant. Therefore, H5a and H5b are partially supported because the moderated mediation effect is statistically significant. This study mainly draws the following conclusions: (1) Social capital can promote entrepreneurial resilience; (2) Both harmonious entrepreneurial passion and obsessive entrepreneurial passion play a partially mediating role in the relationship between social capital and entrepreneurial resilience, that is, social capital can promote entrepreneurial resilience by promoting harmonious entrepreneurial passion and obsessive entrepreneurial passion; (3) Confucian traditional golden-mean thinking positively moderates the relationship between social capital and entrepreneurial resilience, and negatively moderates the effect of obsessive entrepreneurial passion on entrepreneurial resilience.

## Data availability statement

The original contributions presented in the study are included in the article/supplementary material, further inquiries can be directed to the corresponding author.

## Author contributions

TS contributed to developing the theoretical framework, and overall writing of the manuscript. XT contributed to data collection data analysis and editing. Both authors contributed to the article and approved the submitted version.

## Funding

This article was supported by the National Social Science Foundation of China (20CRK004).

## Conflict of interest

The authors declare that the research was conducted in the absence of any commercial or financial relationships that could be construed as a potential conflict of interest.

## Publisher's note

All claims expressed in this article are solely those of the authors and do not necessarily represent those of their affiliated organizations, or those of the publisher, the editors and the reviewers. Any product that may be evaluated in this article, or claim that may be made by its manufacturer, is not guaranteed or endorsed by the publisher.
